#  Genetic variants of calcium sensing receptor gene and risk of colorectal cancer: A case-control study

**DOI:** 10.12669/pjms.35.2.38

**Published:** 2019

**Authors:** Ayat Badr Al-Ghafari

**Affiliations:** 1 *Ayat Badr Al-Ghafari, a) Department of Biochemistry, Faculty of Science, King Abdulaziz University, Jeddah, Saudi Arabia. b) Experimental Biochemistry Unit, c) Cancer and Mutagenesis Unit, King Fahd Medical Research Center, King Abdulaziz University, Jeddah, Saudi Arabia*

**Keywords:** *CASR* gene (rs1801725) variant, *CASR* gene (rs3804594) variant, Colorectal cancer risk, Saudi Arabia

## Abstract

**Objectives:**

To determine the role of two genetic variants, (rs3804594) and (rs1801725), in calcium sensing receptor (*CASR*) gene with colorectal cancer (CRC) risk in patients visited King Abdulaziz University hospital (KAUH) in Jeddah, Saudi Arabia.

**Methods:**

Genomic DNA was extracted, by commercial DNA extraction kit, from whole blood of 100 CRC patients and 124 controls who visited KAUH from January 2016 to September 2016. Then genotype and allele distributions of both variants were determined by PCR-RFLP and DNA sequencing techniques. All statistical analyses were performed by unpaired t-test and *P*-values <0.05 were considered statistically significant.

**Results:**

Data obtained from χ^2^ test showed that intron 4 variant in *CASR* gene was distributed 100% normally in the 224 participants, however, exon 7 variant showed 100% homozygous distribution in the controls; whereas, in CRC patients it was distributed equally into 50% heterozygous and 50% homozygous with no detection for wild type.

**Conclusion:**

Intron 4 variant (rs3804594) in *CASR* gene is not correlated to CRC risk. However, more investigations are needed to elucidate the role of *CASR* gene exon 7 (rs1801725) variant in CRC development as the current results are not definitive.

## INTRODUCTION

The calcium ion (Ca^2+^) is very important in the regulation of several signaling pathways that are contributed to many cellular processes such as proliferation, differentiation, apoptosis, gene expression, and fluid and hormones secretions.^1^ The (Ca^2+^) homeostasis is monitored under the effect of certain G-protein coupled receptor- family C known as calcium sensing receptor (CASR).^2^ The highest expression of CASR is usually seen in the kidney and the chief cells in parathyroid tissues. However, other organs may express CASR on their surfaces such as colon.^3^ In the colon, CASR enhances the differentiation of colonocytes which in turn reduces the formation of neoplasia in the colon.^4^ Moreover, it inhibits fluid and electrolyte secretion, which could potentially serve as a treatment for diarrheal disease.^5^ It may also help the ions of Ca^2+^ to upregulate proteins which modulate duodenal Ca^2+^absorption *in vivo*, although the certain molecular mechanisms behind this physiological process are not clear.^6^ The human *CASR* gene is located on chromosome 3q13.33-q21.1 and contains 11 exons, two promoters and two 5′-untranslated exons (exon 1A and 1B) that yield alternative transcripts, but encoding the same protein.^7^ As many other genes, any genetic variations in the *CASR* gene, either in the form of mutations or single nucleotide polymorphisms (SNPs), can result in a loss or gain of function, which in turn lead to significant alterations in circulating concentrations of calcium that is associated not only with disorders of the parathyroid glands, but also with other conditions such as bone disorders, vascular disorders, and cancer.^1^

Colorectal cancer (CRC) is considered one of the most prevalent cancers with high incidence and morbidity rate worldwide. According to the latest report from the National Cancer Registry (NCR) at King Faisal Specialist Hospital and Research Centre (KFSHRC) in Saudi Arabia, CRC represents the first and the third most common cancer types among males and females, respectively.^8^ Although significant advances in the diagnosis and treatment have been made for CRC patients in Saudi Arabia, the overall 5-year survival rate was (44.6%) for the period 1994-2004 with a high percentage of distant metastasis (28.4%) in patients at the time of presentation and rectal cancer represented (41%) of all colorectal cancer cases diagnosed in 2010.^8^ This poor overall 5-year survival rate is partially due to the lifestyle such as diet and physical activity and partially due to acquired drug resistance.^9^

The CASR is able to respond to a variety of ligands, including polyvalent cations and amino acids. Therefore, any changes of pH and ionic strength that occur in cancer cells may affect the activity of the CASR and subsequently affect its capability to integrate several signaling pathways. Most of the published articles revealed that CASR expression play a protective role in CRC patients through several mechanisms such as binding toxic secondary bile acids and/or ionized fatty acids and neutralizing them in form of insoluble calcium soaps,^10^ or by affecting several signaling transductions such as stimulating cell differentiation, inducing apoptosis and inhibiting proliferation.^11^ To the best of our knowledge, no studies have been conducted to correlate the relation between genetic polymorphisms in *CASR* gene and risk of CRC in Saudi patients. Therefore, this study was aimed to determine the genetic distribution and allele frequency of two SNPs in *CASR* gene in drug-resistant CRC patients that are routinely visiting King Abdulaziz University Hospital (KAUH) to reveal the possible effect, if present, on the pathogenesis of CRC.

## METHODS

In this study, 100 CRC patients and 124 controls were included. The purpose of the research was explained and a written consent of the participants as well as their answers on a questionnaire were obtained. The study was approved by the biomedical ethics unit at faculty of medicine, King Abdulaziz University (KAU) (reference no. 378-17). Several anthropometric measurements such as body weight, height, body mass index (BMI), waist and hip circumference, and waist-to hip-ratio (WHR) were calculated for all participants. All blood samples were drawn into lavender top vacutainers containing anticoagulants (EDTA) and were obtained from oncology clinics at King Abdulaziz University Hospital (KAUH) in Jeddah, KSA from the period January 2016 to September 2016. Genomic deoxyribonucleic acid (gDNA) was extracted from peripheral blood leukocytes in whole blood samples using QIAamp DNA Mini Kit (QIAGEN, Hilden, Germany) following the manufacturer’s instructions. The quality of each extracted DNA sample was assessed by measuring the absorbance at two wavelengths (260 and 280).

### Amplification and genotyping of intron 4 polymorphism (rs3804594) in CASR gene

For a 25 µl PCR reaction, 2 µl genomic DNA (100 ng/µl), 12.5 µl HotStart-IT® FideliTaq™ PCR Master Mix (2X) (catalog no. 71156, Affymetrix, USA), 8.5 µl RNase free water, and 1 µl of (100 pmol/ul) of forward primer: 5’-CAAGGACCTCTGGACCTCCCTTTGC-3’ and reverse primer: 5’-GACCAAGCCCTGCACAGTGCCCAAG-3’) were used. The tubes that contain the PCR mixture were centrifuged at 5000 rpm for 10 min. The PCR thermocycler conditions were as follows: initial denaturation at 94°C for 5 minutes. Followed by 35 cycles of denaturation at 94°C for 30 seconds, annealing at 68°C for 30 seconds, and finally an extension at 72°C for one minute. A final extension step was performed at 72°C for 5 minutes.^12^ The samples were then run on 2% agarose gel to visualize the amplified PCR products (320 bp). The genotyping examination of all PCR samples for intron 4 polymorphism (rs3804594) in *CASR* gene was done by DNA sequencing technique using the sequencer (3130 Genetic Analyzer, serial number: 313001026). The sequencing was done at Centre of Excellence in Genomic Medicine Research (CEGMR) in King Fahd Medical Research Centre (KFMRC), KAU, Jeddah, Saudi Arabia.

### Amplification and genotyping of exon 7 polymorphism (rs1801725) in CASR gene

For a 25 µl PCR reaction, 2 µl genomic DNA (100 ng/µl), 12.5 µl HotStart-IT® FideliTaq™ PCR Master Mix (2X) (catalog no. 71156, Affymetrix, USA), 8.5 µl RNase free water, and 1 µl of (100 pmol/ul) of forward primer:5’-CTGAGCTTTGATGAGCCTCAGAAGGAC-3’and reverse primer: 5’-CACTGATGACAAGCTCTGTGAACTGGA-3’)were used. The tubes that contain the PCR mixture were centrifuged at 5000 rpm for 10 min. The PCR thermocycler conditions were as follows: initial denaturation at 94°C for 5 minutes. Followed by 35 cycles of denaturation at 94°C for one minute, annealing at 63°C for one minute, and finally an extension at 72°C for one minute. A final extension step was performed at 72°C for 5 minutes.^13^The samples were then run on 2% agarose gel with ethidium bromide to visualize the amplified PCR products (269 bp).The genotype distributions of exon 7 polymorphism (rs1801725) in *CASR* gene were determined by restriction fragments length polymorphism (RFLP) procedure. In Eppendorf tube, 10µl of PCR product, 17µl nuclease-free water, 2µl 10x FastDigest green buffer and 1µl of Thermo Scientific FastDigest Hin1I (catalog no. FD0474, Thermo Scientific, USA) were added. Then the mixture was mixed by pipetting gently, and was spin down for few seconds. The incubation was done at 37°C in a heat block for 20 minutes with no inactivation step.

### Statistical analysis

All statistical analyses were performed on GraphPad Prism version 5.00 (San Diego California, USA). A χ^2^ test was used to compare the allele frequency and genotype distribution of each variant following Hardy-Weinberg equilibrium. Unpaired t-test was used to compare between two physical parameters. *P-*values of <0.05 were considered as statistically significant.

## RESULTS

### Anthropometric measurements of study participants

In this study, a total of 224 subjects, n=100 CRC patients and n=124 healthy controls, were included. Unpaired t-test showed that there was highly significant difference between patients and controls in age, weight, and BMI (*P*<0.0001) only as shown in Table-I.

**Table-I:** Comparison of the physical measurements for patients and controls.

Physical measurement	CRC Patients (n=100)	Controls (n=124)	*P*-value
Age (years)	55.73±1.51	40.84±0.78	<0.0001 (HS)
Height (cm)	165.1±1.15	168.0±0.92	>0.05 (NS)
Weight (Kg)	73.2±1.94	83.6±1.44	<0.0001 (HS)
Body Mass Index (Kg/m^2^)	26.8±0.69	29.7±0.49	<0.0001 (HS)
Waist (cm)	100.3±2.43	101.8±1.72	>0.05 (NS)
Hip (cm)	110.0±2.30	108.0±1.58	>0.05 (NS)
Waist to hip ratio	0.917±0.02	0.948±0.01	>0.05 (NS)

All data are represented as mean±standard error of mean, The *P*-values were calculated by unpaired t-test, HS: highly significant, NS: no significant difference.

### Genotype distribution and allele frequency of intron 4 polymorphism (rs3804594) in CASR gene

**Fig.1:**
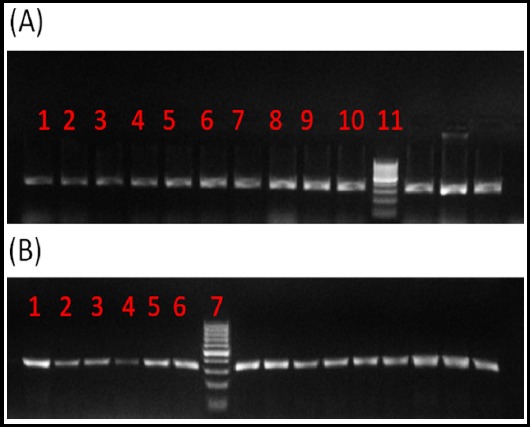
Genotyping of intron 4 polymorphism (rs3804594) in *CASR* gene. (A) Showed the gel electrophoresis for the CRC patients, where lanes (1-10) represents the PCR products for P1-P10 (320 bp) and lane 11 represents the DNA ladder 100 bp). (B) Showed the gel electrophoresis for the healthy controls, where lanes (1-6) represents the PCR products for C1-C6 (320 bp) and lane 7 represents the DNA ladder 100 bp).

The amplified fragments showed a size of 320 bp. To determine the polymorphic genotypes, the PCR products were analyzed with DNA sequencing technique.The normal (TT) genotype produced only one band of size 320 bp. The heterozygous (TC) genotype produced three fragments of sizes 60, 260, and 320 bp, and the homozygous (CC) genotype produced two fragments of sizes 60 and 260 bp (Fig.1). The DNA sequencing analysis of SNP (rs3804594) revealed that all subjects showed 100% wild type with no reported homozygous or heterozygous genotypes. Therefore, this SNP is rare in the study population and therefore, cannot be correlated with the CRC risk.

### Genotype distribution and allele frequency of exon 7 polymorphism (rs1801725)in CASR gene

**Fig.2:**
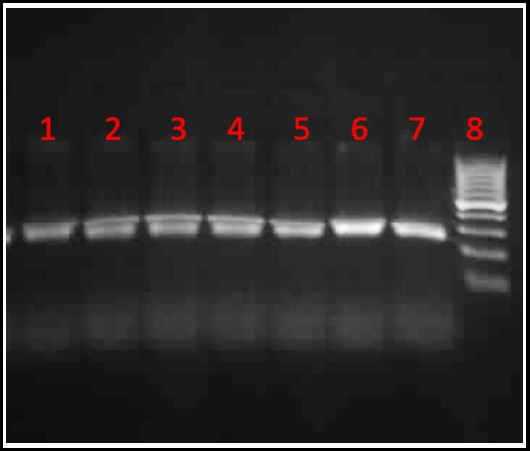
Genotyping of exon 7 polymorphism (rs1801725) in *CASR* gene. This gel represents the PCR products after digesting with Hin1I enzyme. Lanes (1-5) showed heterozygous genotypes with three bands of sizes (28, 241, and 269 bp). Lane 6 showed homozygous genotype with one band of size (269 bp). Lane 8 represents the DNA ladder (100 bp).

The amplified fragments showed a size of 269 bp. To determine the polymorphic genotypes, the PCR products were digested with Hin1I enzyme.The normal (GG) genotype produced two bands of sizes 128 and 241 bp. The heterozygous (GT) genotype produced three fragments of sizes 28, 241, and 269 bp, and the homozygous (TT) genotype produced only one fragment of size 269 bp(Fig.2). The genotypic frequencies of the patients were 0% (n=0) normal (GG), 53% (n=53) heterozygous (GT), and 47% (n=47) homozygous (TT). The frequency of G allele was (26.5%) and T allele was (73.5%). Genotype distribution for CRC patients was out Hardy-Weinberg equilibrium (Goodness of fit χ^2^=16.82, degree of freedom (DF)=1, *P*<0.05). In controls, the results showed 0% (n=0) normal (GG), 0% (n=0) heterozygous (GT), and 100% (n=124) homozygous (TT). The frequency of G allele was (0%) and T allele was (100%). Genotype distribution for the controls was out of Hardy-Weinberg equilibrium (Goodness of fit χ^2^ =122, DF=1, *P*<0.05). The calculation of the risk and odds ratios, Fisher’s *P*-value and 95% confidence interval by the 2x2 contingency table were impossible since no patients or controls showed normal (GG) genotype. Therefore, it was statistically difficult to correlate the genotypes distribution of this SNP with increased or decreased risk of CRC in the study population.

## DISCUSSION

CRC is one of the most prevalent types of cancers and a major cause of death globally. In Saudi Arabia, although many advances have been made in the diagnosis and treatment of CRC, this cancer type still ranks the first and the third most common malignancies among males and females, respectively.^8^ Like other types of cancers, CRC resulted from an interaction between several factors such as chemical carcinogens, genetic factors, inflammatory diseases and diet. Diet plays a major role in colorectal tumorigenesis. Several epidemiological studies showed an inverse correlation between calcium intake and the risk of tumor development.^14^,^15^ Calcium performs several important physiological processes such as cell signaling, blood clotting, muscle contraction, nerve function, enzymes activation, ions transportation across the cellular membrane, and neurotransmitters communications with other cells.^16^ It is known that calcium performs its function through binding to special G protein -coupled receptor known as calcium sensing receptor (CASR). In cancer, CASR expression can be reduced, lost, or become activated. Moreover, the calcium signaling may differ depending on the type of cancer, stage, grade, and other factors such as in colon and parathyroid cancers (it acts as tumor suppressor), whereas, in breast and prostate cancers (it acts as an oncogene).^17^-^20^

Around one hundred and twelve mutations (98 missense, 6 nonsense, 8 insertion and/or deletion, and one splice mutation) have been described in the *CASR* mutation database and were tested for their contributions to several diseases including diabetes mellitus, kidney diseases, and parathyroid adenoma.^12^,^21^,^22^ However, fewer researches were conducted on CRC patients. Therefore, there is an urgent need for a better understanding of how individual ligands affect CASR-mediated signaling, and how this will in turn benefit the development of novel pharmaceutical therapies targeting the CASR in CRC. To the best of our knowledge, there are no conducted studies that determine the effect of genetic variants in *CASR*gene in CRC patients in Saudi Arabia. Therefore, in this study, two SNPs in *CASR* gene were determined in CRC patients and control subjects to reveal their implications in CRC risk. In the current study, the analysis by PCR-RFLP and DNA sequencing revealed that variant (rs3804594) in intron 4 showed a 100% wild type in our study population. Neither heterozygous nor homozygous were determined in either CRC patients or controls. On the other hand, interestingly, results showed that variant (rs1801725) in exon 7 showed 0% normal genotype and 50% for the both genotypes (heterozygous and homozygous) in CRC patients, whereas, in controls, the results showed 100% homozygous genotype with no distribution for normal or heterozygous genotypes. Unfortunately, in both SNPs, the correlation between genotypes distribution and allele frequency for both SNPs and CRC risk was difficult to be calculated since no normal genotype cases were recorded, therefore, risk and odds ratios were not calculated by chi-square test.

Regarding the association of variant (rs3804594) in intron 4 with cancer implications, less is known about the effect of this SNP with cancer risk as most of the published results studied other SNPs in different regions (introns or exons) rather than SNP (rs3804594). In the current study, (rs3804594) was found to be normally distributed in the study population therefore, has no effect on CRC risk. However, variant (rs3804594) was found to be highly correlated with the risk of diabetes mellitus Type-2 and diabetic nephropathy.^12^

On the other hand, regarding the association of variant (rs1801725) with cancer risk, in agreement with our results, Wang et al. showed that the mean calcium levels were higher in breast cancer patients compared to controls (*P*< 0.001) due to inactivating polymorphisms at rs1801725.^23^ Moreover, a research performed on Iranian CRC patients showed that *CASR* gene (rs1801725) variant is not a genetic contributor to CRC risk in the Iranian population.^13^ In another case-control study that was conducted to explore the possible association between (rs1801725) variant in *CASR* gene and CRC risk, they found no significant difference for this variant in both genotype and allele frequencies between CRC and the controls.^24^ Variant (rs1801725) was found to be significantly associated with clinical outcome in patients with stage four and the histological subgroup of undifferentiated neuroblastomas. Moreover, patients harboring this polymorphism had significantly lower overall and event-free survival rates than those who were homozygous for the most common allele.^25^

## CONCLUSION

To the best of our knowledge, this is perhaps the first study that determined the effect of genetic variants in *CASR* gene in CRC patients in Saudi Arabia. The analysis by PCR-RFLP and DNA sequencing revealed that none of the two variants are correlated with CRC risk in Saudi patients.However, the analysis are recommended to be performed on larger population and to compare the finding of this study with the recent published data whenever it is available. Moreover, immunohistochemistry technique can be used to evaluate the expression of the CASR in the tissues samples from CRC patients with different stages and correlate them with disease progression.
